# GiniClust3: a fast and memory-efficient tool for rare cell type identification

**DOI:** 10.1186/s12859-020-3482-1

**Published:** 2020-04-25

**Authors:** Rui Dong, Guo-Cheng Yuan

**Affiliations:** 1 Department of Pediatric Oncology, Dana-Farber Cancer Institute, Boston, MA 02215 USA; 2 Boston Children’s Hospital, Boston, MA 02115 USA; 3 Harvard Medical School, Boston, MA 02115 USA

**Keywords:** Scalability, Rare cell identification, Gini index, Single cell RNA-seq

## Abstract

**Background:**

With the rapid development of single-cell RNA sequencing technology, it is possible to dissect cell-type composition at high resolution. A number of methods have been developed with the purpose to identify rare cell types. However, existing methods are still not scalable to large datasets, limiting their utility. To overcome this limitation, we present a new software package, called GiniClust3, which is an extension of GiniClust2 and significantly faster and memory-efficient than previous versions.

**Results:**

Using GiniClust3, it only takes about 7 h to identify both common and rare cell clusters from a dataset that contains more than one million cells. Cell type mapping and perturbation analyses show that GiniClust3 could robustly identify cell clusters.

**Conclusions:**

Taken together, these results suggest that GiniClust3 is a powerful tool to identify both common and rare cell population and can handle large dataset. GiniCluster3 is implemented in the open-source python package and available at https://github.com/rdong08/GiniClust3.

## Background

The rapid development of single cell technologies has greatly enabled biologists to systematically characterize cellular heterogeneity (see reviews [1–4]). While many methods have been developed to identify cell types from single cell transcriptomic data [5–7], most are designed to identify common cell types. As the throughput becomes much higher, it is also of considerable interest to specifically identify rare cell types. Several methods have been developed [8–13]; however, existing methods are not scalable to very large datasets. Considering the fact that atlas-scale datasets may contain hundreds of thousands or even millions of cells [5, 14–16], there is an urgent need to develop faster method for rare cell type detection.

In previous work, we developed GiniClust to identify rare cell clusters, using a Gini-index based approach to select rare cell-type associated genes [11]. Recently, we extended the method to identify both common and rare cell clusters, using a cluster-aware, weighted ensemble clustering approach [12]. These methods have been used to analyze datasets containing up to 68,000 cells. Here we have further optimized the algorithm so that it can be efficiently used to analyze dataset containing over one million cells. By using a real single-cell RNA-seq dataset as an example, we show that this new extension, which we call GiniClust3, can efficiently and accurately identify both common and rare cell types.

## Implementation

### Details of GiniClust3 pipeline

**Fig. 1:**
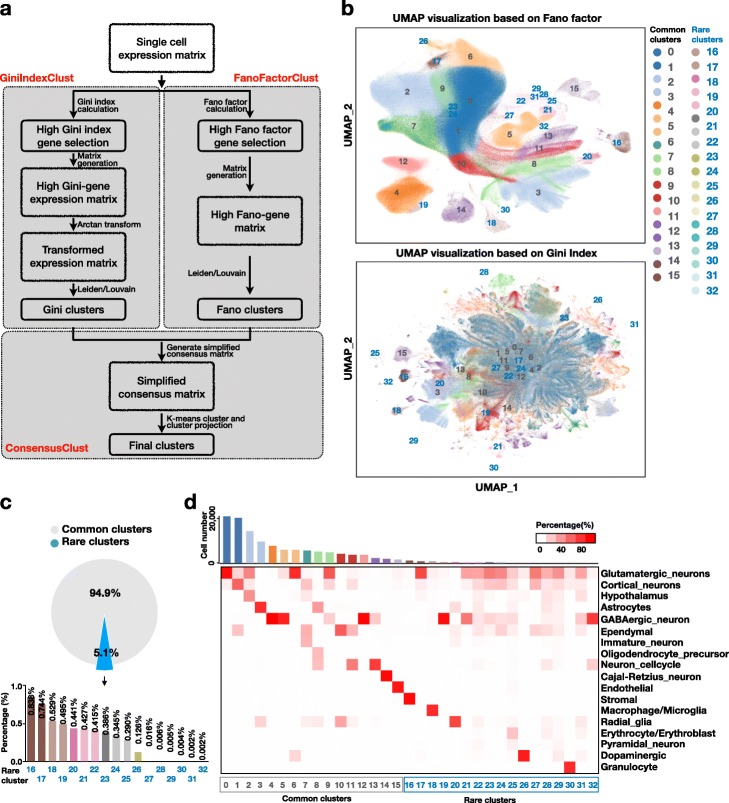
Analysis of mouse brain dataset with more than one million cells. **a** An overview of the GiniClust3 pipeline. Input single-cell expression matrix is clustered based on features selected by Gini index (GiniIndexClust) and by Fano factor (FanoFactorClust), respectively. The results are then integrated using a cluster-aware, weighted consensus clustering algorithm (ConsensusClust). **b** UMAP visualization of the gene expression patterns based on Fano-factor (top) and Gini index (bottom) selected features, respectively. Consensus clustering results are indicated by different colors. **c** The proportion of rare cell cluster in entire population. **d** Heatmap of cell type mapping of common and rare clusters from scMCA analysis. Bar plot in the top indicates the cell number for each cluster

The overall strategy is similar to GiniClust2 [12]. The implementation of each step is optimized to improve computation and memory efficiency (Fig. 1a). Compare with GiniClust2, there are two major changes. First, we used Leiden, which were suitable for large datasets, to replace DBSCAN for the clustering step. Second, we generated consensus matrix based on cluster level of Gini and Fano cluster results, instead of cell level. Both changes could highly increase the computational efficiency. The details of the GiniClust3 pipeline are as follows.

#### Step 1: clustering cells using Gini index-based features

- *Gini index calculation and normalization.* After data pre-processing, the Gini index for each gene is calculated as twice of the area between the diagonal and Lorenz curve, as described before [11]. The range of Gini index values is between 0 to 1. Then, Gini index values are normalized by using a two-step LOESS regression procedure as described before. Genes with Gini index value ≥0.6 and *p* value < 0.0001 are labeled as high Gini genes and selected for further analysis.

b. *Cell cluster identification by Leiden algorithm*. In previous versions [11, 12], DBSCAN was used for clustering. While DBSCAN is effective for identify rare cell clusters, this method is both time and memory consuming. In GiniClust3, we replace DBSCAN with the Leiden clustering algorithm [17], which is known for improved numerical efficiency. Alternatively, users can also select the Louvain clustering algorithm [18] by setting “method = louvain”. The neighbor size we set in Gini index-based clustering of mouse brain single-cell dataset is 15 (neighbors = 15). Lower threshold for neighbor size to efficiently identify rare clusters in smaller datasets is recommended (default value = 5).

#### Step 2: clustering cells using Fano factor-based features

Highly variable genes are identified by using Scanpy. These genes are used to identify common cell clusters by using principal component analysis (PCA) followed by Leiden or Louvain clustering, using the default settings in Scanpy [7]. The neighbor size we set in Fano factor-based clustering of mouse brain single-cell dataset is 15 (neighbors = 15).

#### Step 3: combining the clusters from steps 1 and 2 via a cluster-aware, weighted consensus clustering approach effectively

The weighted consensus clustering method is described before [12] with modifications. Connectivity of cells in different cluster results (*P*^*G*^ and *P*^*F*^) are calculated. To improve computational efficiency, we kept one cell to represent cells with same Gini and Fano cluster results. Thus, the computational efficiency is associated with Gini and Fano cluster numbers rather than cell numbers. Then, we calculate the consensus matrix based on these *n* cells from different Gini and Fano clusters. If two cells are clustered in the same group, the connectivity is 1, otherwise the connectivity is 0 (formula (a)). We set the cell-specific weights for the Fano factor-based clusters *w*^*F*^ as a constant value *f’* while the cell-specific GiniIndexClust weight *w*^*G*^ are determined as a logistic function of the size of cluster containing the particular cell (formula (b)), where *x*_*i*_ is the proportion of the GiniClust cluster for cell *i*, *μ’* is the rare cell type proportion at which GiniClust and Fano factor-based clustering methods have approximately the same ability to detect rare cell types, and *s’* represents how quickly GiniClust loses its ability to detect rare cell types above *μ’*.

a $$ {M}_{ij}\left({P}^G\right)={\displaystyle \begin{array}{c}\Big\{\begin{array}{c}1,\left(i,j\right)\in {C}_k\left({P}^G\right)\\ {}0, otherwise\end{array},\mathrm{i},\mathrm{j}\in \left(1,\cdots, \mathrm{n}\right)\\ {}\kern0ex and{M}_{ij}\left({P}^F\right)=\Big\{\begin{array}{c}1,\left(i,j\right)\in {C}_k\left({P}^F\right)\\ {}0, otherwise\end{array}\operatorname{}\operatorname{}\end{array}},\mathrm{i},\mathrm{j}\in \left(1,\cdots, \mathrm{n}\right) $$

b $$ \tilde{w}_{i}^G=1-\frac{1}{1+{e}^{-\frac{x_i-{\mu}^{\prime }}{s^{\prime }}}} $$

The cell pair-specific weights were firstly defined as formula (c). Then, after normalization of the *w*^*F*^ and *w*^*G*^ (formula (d)), the consensus value was calculated based on the weight (${w}_{ij}^G$ and ${w}_{ij}^F$) and connection (*M*_*ij*_(*P*^*G*^) and *M*_*ij*_(*P*^*F*^)) (formula (e)).

c $$ \tilde{w}_{ij}^G=\max \left(\tilde{w}_{i}^G,\tilde{w}_{j}^G\right) and\tilde{w}_{ij}^F=\tilde{w}_{i}^F $$

d $$ {w}_{ij}^G=\frac{\tilde{w}_{ij}^G}{\tilde{w}_{ij}^G+\tilde{w}_{ij}^F} and{w}_{ij}^F=\frac{\tilde{w}_{ij}^F}{\tilde{w}_{ij}^G+\tilde{w}_{ij}^F} $$

e $$ \overline{M_{ij}}={w}_{ij}^G{M}_{ij}\left({P}^G\right)+{w}_{ij}^F{M}_{ij}\left({P}^F\right) $$

k-means clustering is applied to the consensus matrix $\overline{M_{ij}}$, then the results are easily converted back to single-cell level clustering. Finally, clusters with cell population < 1% are considered as rare clusters.

### Data source and pre-processing of the data

A mouse brain single-cell RNA-seq dataset was downloaded from 10X genomics website: (https://support.10xgenomics.com/single-cell-gene-expression/datasets/1.3.0/1M_neurons). This dataset contains 1.3 million cells obtained from cortex, hippocampus and ventricular zones of E18 mice. Raw data was pre-processed by using Scrublet [19] (version 0.2.1) to remove doublets with default setting. The resulting data was further filtered to remove genes expressed in fewer than ten cells and cells expressed fewer than 500 genes. A total number of 1,244,774 cells and 21,493 genes passed this filter were retained for further analysis. Raw UMI counts were normalized by Scanpy [7] with the following parameter setting: sc.pp.normalize_per_cell (counts_per_cell_after = 1e4).

## Results

Compared with GiniClust2, we did two major modifications to optimize the performance. First, clustering method which consumes time and memory is replaced with method suitable for large scale dataset. Second, we speed up GiniClust3 by generating consensus matrix in cluster level rather than cell level. Both the modifications could highly increase the speed and reduce the memory consumption of GiniClust3.

To test the utility of GiniClust3, we applied the method to analyze a public single-cell RNA-seq dataset containing 1.3 million single cells obtained from three regions in the mouse brain (see Implementation for details). After filtering out lowly-expressed genes and poor-quality cells (such as those likely to be doublets), a 1,244,774 cell-by-21,494 gene count matrix was left for further analysis. We next sought to characterize the identities of cell populations by using GiniClust3. A total number of 16 common and 17 rare cell clusters (cell population < 1%) were identified (Fig. 1b, S1a), with the smallest cluster containing only 21 cells (cell population = 0.002%) (Fig. 1c and Table S1). The total time of cluster identification for both common and rare cell took ~ 7-h time, and 103G memory on a Xeon E5–2683 with 56 threads and 640GB memory server, indicating GiniClust3 is suitable for analyzing very large datasets.

To annotate these cell clusters, we mapped each cluster to mouse cell atlas (MCA) [14] by using the scMCA algorithm [20]. Ten of the sixteen common clusters (cluster 0, 1, 4, 5, 6, 9, 12, 13, 14 and 15) were mapped to specific cell types in MCA with expected abundance. These include glutamatergic neurons, astrocytes, GABAergic neuron, ependymal, cell cycle neuron, cajal-retzius neuron and endothelial (Fig. 1d). For example, cluster 0 is mapped to glutamatergic neurons, which are known to be the most abundant neuronal cell type [21, 22]. Eight of the seventeen rare clusters (cluster 16, 17, 18, 19, 20, 26, 30 and 32) can be mapped to previously annotated cell types. These include stromal, glutamatergic, macrophage/microglia, radial glia, dopaminergic, granulocyte and GABAergic neuron. Of note, GiniClust3 was able to identify granulocyte cells (cluster 30), even though they represent a tiny fraction (55 out of 1,244,774 cells, 0.004%) of the cell population, indicating the sensitivity of GiniClust3 is very high.

We then systematically evaluate the time and memory consumption in different scales, we randomly subsampled 1.3 million mouse brain scRNA-seq dataset, range from 5 K to 1 M cells. The time and memory consumption scale almost linearly with cell number, as the regression slope is close to 1 in both cases (Fig. S1b, slope = 1.08 for running time; Fig. S1c, slope = 0.92, for memory usage). To evaluate the robustness of GiniClust3, we repeated the analysis using randomly subsampled data. To this end, 50% of the cells were randomly selected from common clusters (≥1%). Since our main focus was to identify rare cell clusters, the cells assigned to these rare clusters (< 1%) identified above were all retained. By repeating this subsampling method for 10 times and applying GiniClust3 to the subsampled datasets, we found most of the clusters in subsampled datasets are consistent with the original ones, the median Normalized Mutual Information (NMI) is 0.81 (Fig. S1d). Taken together, these analyses show that GiniClust3 is a sensitive, accurate and efficient clustering method that can be used in many applications.

## Conclusions

With the technological development and protocol improvement, the scaling of single-cell RNA-seq is increasing in an exponential way [23], providing a great opportunity to identify previously unrecognized rare cell types. We have shown that GiniClust3 is an accurate and highly scalable method for detecting rare cell types from large single-cell RNA-seq datasets. GiniClust3 could identify both common and rare cell population and handle large dataset containing more than one million cells in an effective way. This property is important to comprehensively identify cell types in large datasets and may be particularly useful for atlas datasets in future.

### Availability and requirements

Project name: GiniClust3

Project home page: https://github.com/rdong08/GiniClust3

Operating system: Platform independent

Programming language: python

Other requirements: python 3.0 or higher

License: GPL

Any restrictions to use by non-academics: License needed

## Supplementary information

**Additional file 1: Figure S1.** a A gene expression heatmap showing the top differentially expressed genes for each cell cluster identified from the mouse brain single-cell RNA-seq dataset. b Time consumption of GiniClust3 in subsampled data with varying cell numbers. c Memory consumption of GiniClust3 in subsampled data with varying cell numbers. d Normalized mutual information (NMI) values quantifying the agreement between GiniClust3 clustering results from randomly selected subsamples of the mouse brain dataset. 10 random subsamples were generated for which the results are compared here.

**Additional file 2: Table S1.** Clusters, marker genes and cell mapping results using scMCA in mouse brain dataset.

## Data Availability

The mouse brain 10X sequencing data is available from 10X genomics website: (https://support.10xgenomics.com/single-cell-gene-expression/datasets/1.3.0/1M_neurons).

## Abbreviations

DBSCAN: Density-Based Spatial Clustering of Applications with Noise
MCA: Mouse Cell Atlas
PCA: Principal Component Analysis
UMAP: Uniform Manifold Approximation and Projection
NMI: Normalized Mutual Information
