# Supplementary figures and images for: GiniClust3: a fast and memory-efficient tool for rare cell type identification
Source: BMC Bioinformatics. 2020 Apr 25;21:158. doi: 10.1186/s12859-020-3482-1 (PMC7183612; doi:10.1186/s12859-020-3482-1)

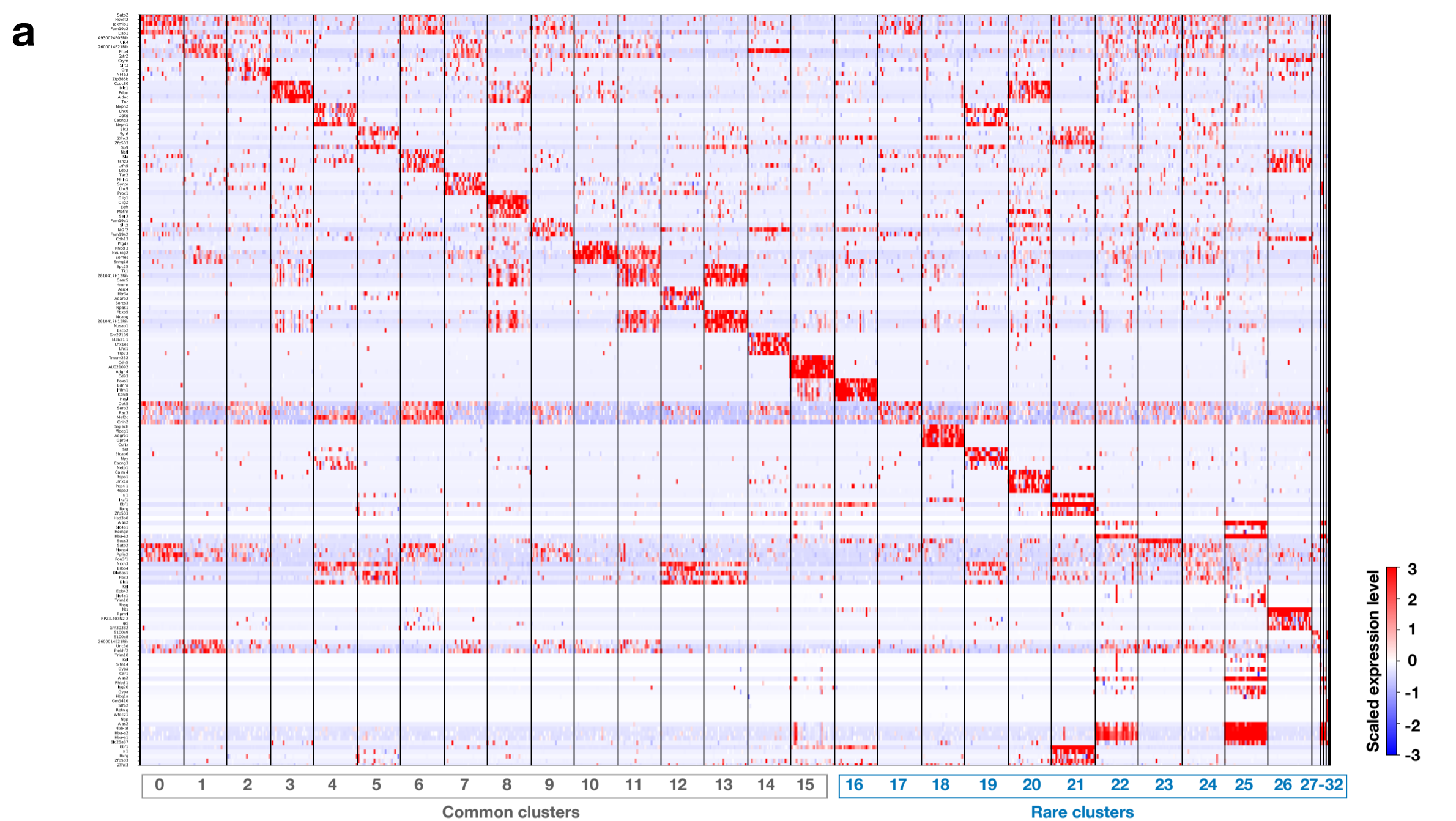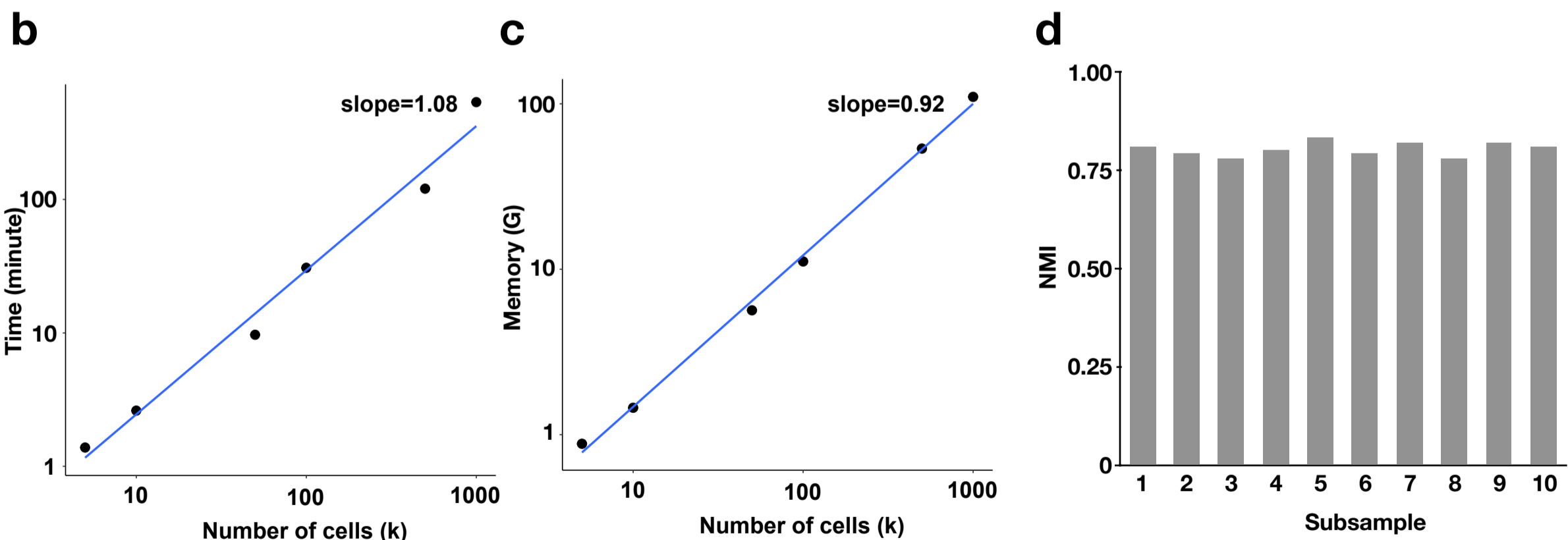

**Supplementary Fig. 1**

Supplement: Supplementary file 1 — Additional file 1: Figure S1. a A gene expression heatmap showing the top differentially expressed genes for each cell cluster identified from the mouse brain single-cell RNA-seq dataset. b Time consumption of GiniClust3 in subsampled data with varying cell numbers. c Memory consumption of GiniClust3 in subsampled data with varying cell numbers. d Normalized mutual information (NMI) values quantifying the agreement between GiniClust3 clustering results from randomly selected subsamples of the mouse brain dataset. 10 random subsamples were generated for which the results are compared here. [file 12859_2020_3482_MOESM1_ESM.pdf]
